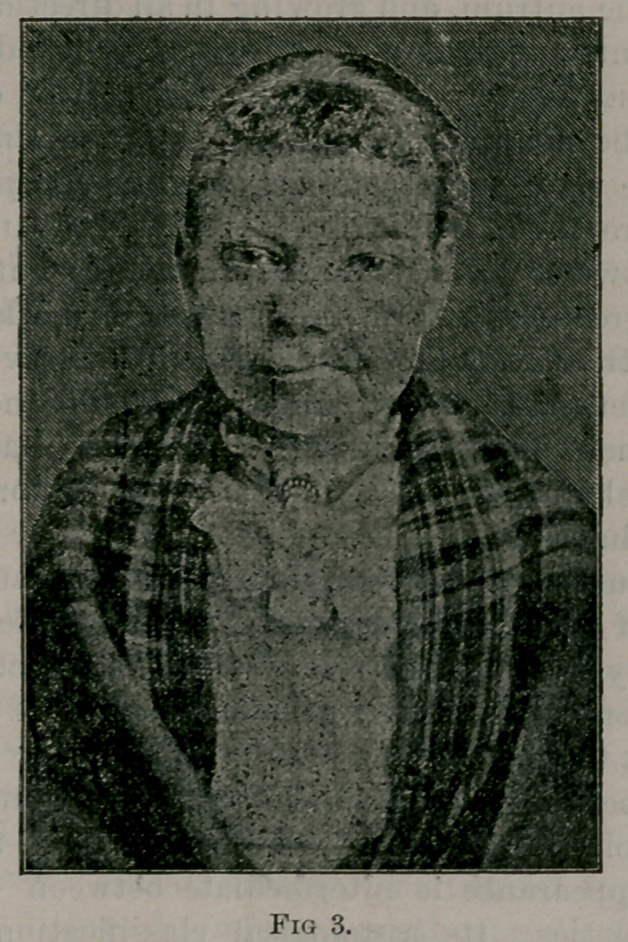# Cases in Surgery

**Published:** 1884-07

**Authors:** T. M. McIntosh

**Affiliations:** Thomasville, Ga.


### CASES IN SURGERY.*

BY T. M McINTOSH, M. D., THOMASVILLE, GA.

### CASE 1.—REMOVAL OF SUPERIOR MAXILLA FOR TUMOR.

Alice M——, white, age 11 years, Brooks county, Ga., noticed four years ago an enlargement on right superior maxilla, situated above the alveolar border, beginning without any known cause, and continuously growing without pain either in the tumor or surrounding parts.

After twelve months, the tooth immediately beneath the swelling was removed, with an idea that it might possibly be the cause for the enlargement or aggravate its growth, but instead of retarding had the apparent effect of increasing its rapidity. It has now attained an immense size and produces a hideous deformity and great discomfort from its size, putting the cheek, by its outward growth, greatly upon the stretch and thinning it very much. Internally it has extended beyond the articulation of the palate bones, posteriorly to the pterygoid process of the sphenoid bone, anteriorly to the canine tooth of the opposite maxilla, and inferiorly it had pressed so much upon the lower maxilla as to cause a partial absorption of its alveola border, the teeth in this part being only rudimentary. It had so widely stretched the mouth that alimentation was very difficult, even with liquid food.

To the touch, the tumor was resilient, semi-elastic, but firm, of a consistence somewhat between a pure fibroid and a medullary tumor It bled readily from a rough touch from the palatine portion, and had done so previously a good deal at times. Over its surface numerous arteries could be felt pulsating, but all were superficial, seemingly, and circulation in them arrested by pressure of the carotid.

---

*Reported at the annual meeting of the Medical Association of Georgia, held at Macon, April, 1884.

FIG. 1.

FIG. 2.

The child was rather pale and anæmic from occasional loss of blood, but principally from malnutrition, because of difficulty of eating little else than liquid foods.

With the aid of Drs. Taylor, Dekle, Bruce, Welington, Arnold, Hopkins and J. H. and J. B Coyle, the operation was done by making incision, beginning at a point on the upper lip, immediately below the left alae nasi, extending upward, alongside the nose to near the inner angle of the eye, thence at right angles to the zygomatic arch. The flap thus formed, and the nose as far as its cartilage, were dissected up, and the bony parts divided with cutting forceps from the left canine tooth, to a point on the right palate bone one-fourth of an inch from its articulation with its fellow. The nasal, orbital and molar connections were sawed through, when with a firm downward pressure, the tumor was removed.

Throughout the operation the circulation in the carotid was controlled by digital compression, thus enabling me to finish the operation with a slight loss of blood. It was necessary to ligate but two arteries—the facial and transverse facial. There was, during the operation, marked failure of the pulse, which required the prompt administration of hypodermics of whisky and ammonia to overcome.

Incidentally, in regard to hypodermic medication, I will mention that these injections produced two abscesses, one of which resulted in a large slough as deep as the muscular tissue. This was due, in

FIG 3.

my opinion, to the ammonia, as I have never had it to occur, when I used the whisky alone, which I have done quite frequently.

The shock of the operation was considerable, from which there was not complete reaction for several hours, when it took place fully and not excessively. No other unfavorable or notable symptoms took place during the entire history to recovery.

The wound was well packed with cotton wetted with solution of carbolic acid and water, which was daily removed, and the wound thoroughly washed with warm water by means of a Davidson's syringe. Morphine was given as required. Milk punches and other liquid food the method of alimentation. The operation was done December 8th, 1883; the patient went home on January 4th, 1884.

The accompanying photographs show very well the appearance prior to and after removal.

Fig. 1, front view, fig. 2, side view, fig. 3, appearance of patient three months from time of operation.

The tumor originated within the substance of the bone, or possibly within the autrum, and growing in all directions, absorbed by pressure the entire alveola portion, where relieved from pressure, grew more rapidly externally than in that portion confined within the cavity of the autrum. Frequently it occurs that tumors originating in bone have an osseous case to form *pari passu* with their growth, and are thus completely surrounded by an osseous envelope. This, however, is only incased in a thin lamella of bone in that portion growing within the antral cavity, which constitutes about one-fourth of its bulk. From within this cavity a part of the tumor had extended backward, under the floor of the orbit, as far as the optic foramen. When removed, the tumor had a rather firm, semi-elastic feel, and presented on section, a uniform, whitish color, and somewhat fibrous structure.

The first bicuspid tooth is hanging by a fleshy attachment with only a trace of its alveolus present. No other evidence of teeth, or of its maxillary origin are present, owing to the fact that the tumor began and absorbed the alveoli prior to the time of the irruption of the permanent teeth.

The tumor belongs to the connective tissue group of new formations of heterologous structure, of the variety—Sorcomata. Its microscopic appearance is intermediate between the fibrous and medullary varieties. Its histological classification I have not determined, not having submitted it to the microscope, but it is probably a giant celled sorcoma. Clinically, in the majority of cases they do not recur, though, as with many forms of sorcomata, this is liable to take place either in the original site of the tumor, or in some internal organ.

In forming a prognosis, the slowness or rapidity of growth, the presence or absence of pain, the consistence, location, vascularity, age of patient, are equally as reliable guides as histological structure, which bears no universal, specific relation to the clinical features of tumors.

Paget has called this kind of tumor myeloid, because of resemblance to brain tissue; Sibert fibro-plastic, because of its constitution of fibre cells. It is the central osteo-sorcoma of Billroth, though it has none of the cystic characters of some osseous tumors described by that author.

In this case I am hopeful that no recurrence will take place, as the removal was complete, and it does not occupy a position near to the extreme end of the sorcomata where malignancy is the rule.

## Figures and Tables

**Fig. 1. f1:**
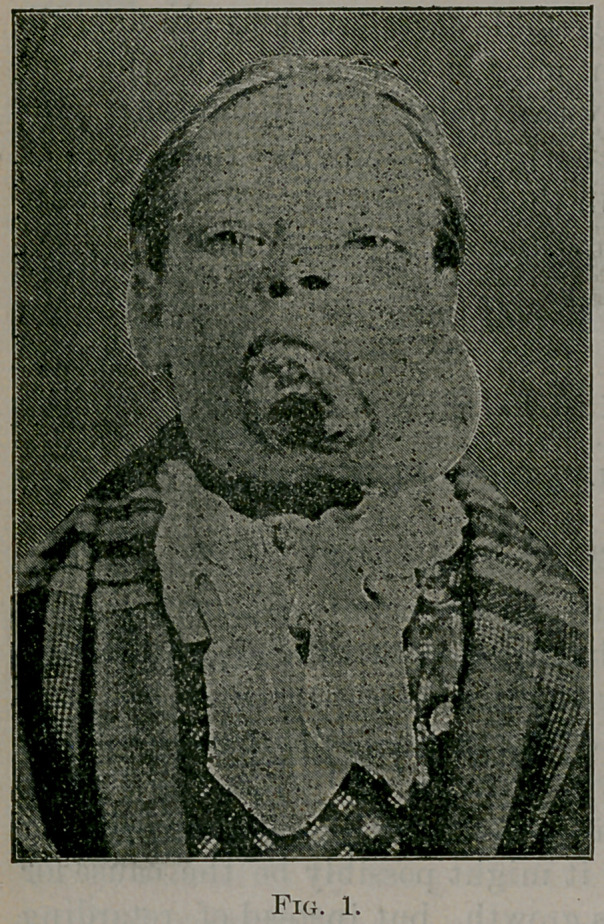


**Fig. 2. f2:**
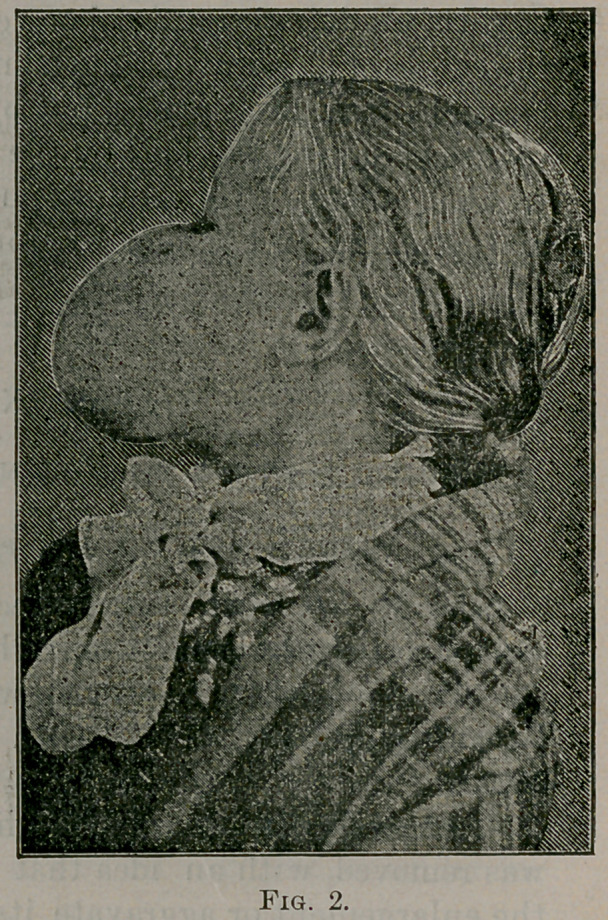


**Fig. 3. f3:**